# Preampullary Duodenal Web Simulating Gastric Outlet Obstruction

**Published:** 2013-01-01

**Authors:** Ramnik V Patel, Hemant Kumar, Bharat More

**Affiliations:** * Department of Pediatric Surgery, UCLH and GOSH, London, UK; 1 Department of Pediatric Surgery, Leicester Royal Infirmary, Leicester, UK

**Dear Sir**

Duodenal atresia usually presents with bilious vomiting. Presentation of duodenal atresia with non-bilious emesis can simulate gastric outlet obstruction [1-5]. Herein, we highlight technical aspects of identification of windsock anomaly of the duodenum that often deceives the operating team.

A 5-day-old female neonate presented with persistent non-bilious projectile vomiting since birth. Baby girl was born at term by normal vaginal delivery. Antenatal scans at 12 and 20 weeks of pregnancy were reported as normal. Mother had polyhydramnios in late pregnancy. Baby was discharged home the same day of birth. The baby however developed non-bilious projectile emesis on the same day after feeding attempts. The baby was initially treated as a case of mucous gastritis and gastro-oesophageal reflux without any improvement. 

Laboratory tests showed metabolic alkalosis and acute renal failure. Capillary blood gas showed Ph 7.56, base excess -22 and HCO3 was 44 mmol/L, potassium 3 mmol/L and chloride 85 mmol/L. Plain x-ray abdomen showed gas in the stomach only (Fig. 1a). She was resuscitated, started on antibiotics and transferred to us. On arrival, baby was dehydrated but well perfused. There was visible left to right peristalsis in the epigastric region and the rest of the abdomen was scaphoid and soft. Pyloric tumour was not palpable during test feed. Complete blood count was normal. Biochemical profile showed sodium 133 mmol/L, potassium 3.6 mmol/L, urea 11.9 mmol/L, creatinine 193 mmol/L, total bilirubin 287 mmol/L with conjugated fraction being 12. Her renal functions gradually improved with adequate hydration.

Ultrasound scan of the cranium was normal. Abdominal ultrasound scan showed fluid filled distended first part of duodenum and collapsed distal bowel loops with an appearance of luminal structure like a diaphragm in the lumen of the duodenum. She underwent upper gastrointestinal contrast study and the pilot film showed double bubble appearance (Fig. 1b). There was severe gastro-oesophageal reflux up to thoracic inlet level (Fig. 1c). The stomach was hypertrophied and dilated up to first part of duodenum which was dilated (Fig. 1d). Lateral view demonstrated clear windsock deformity but it was difficult to see the rim of the attachment of the diaphragm on the duodenal wall (Fig. 1e).

At exploration, there was no discontinuity of the duodenum externally and there was no visible or palpable rim of the attachment of the diaphragm externally. The first and second parts of duodenum were uniformly dilated and it was difficult to determine the exact site of the obstruction. A wide bore orogastric tube was passed into the stomach and pushed tenting at the windsock deformity. This led to demonstration of a faint indentation at the junction of the first and second part of duodenum.

A longitudinal incision centered on the indentation was made on the anterior duodenal wall and a complete mucosal duodenal diaphragm without any hole was identified. A distal windsock deformity was clearly evident (Fig. 2A and 2B).

**Figure:**
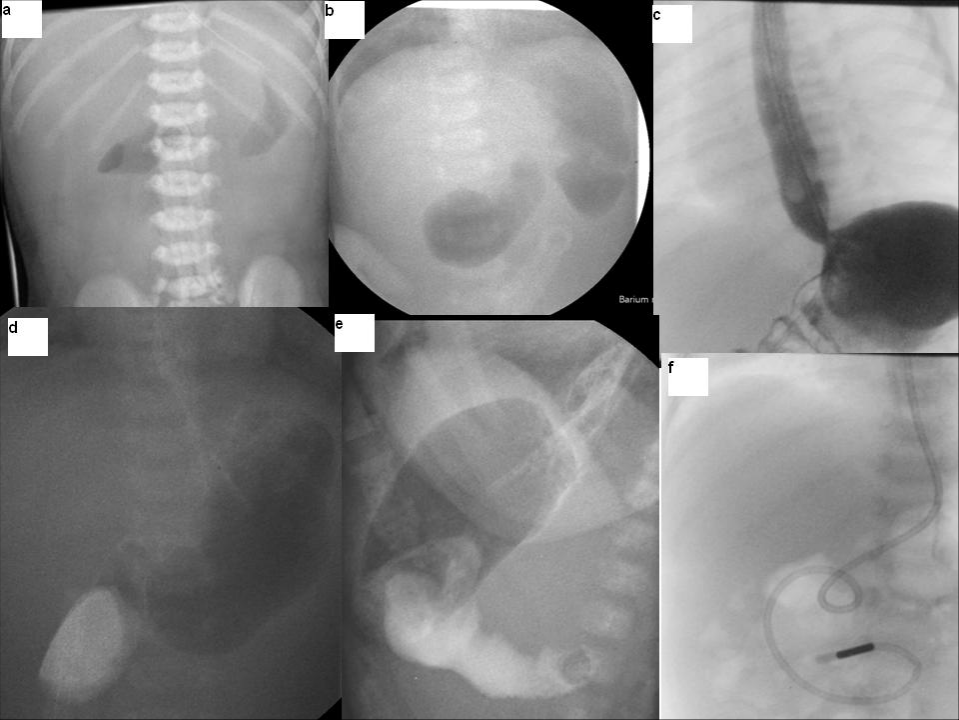
Figure 1: Plain and contrast imaging in the pre and post operative period.

**Figure:**
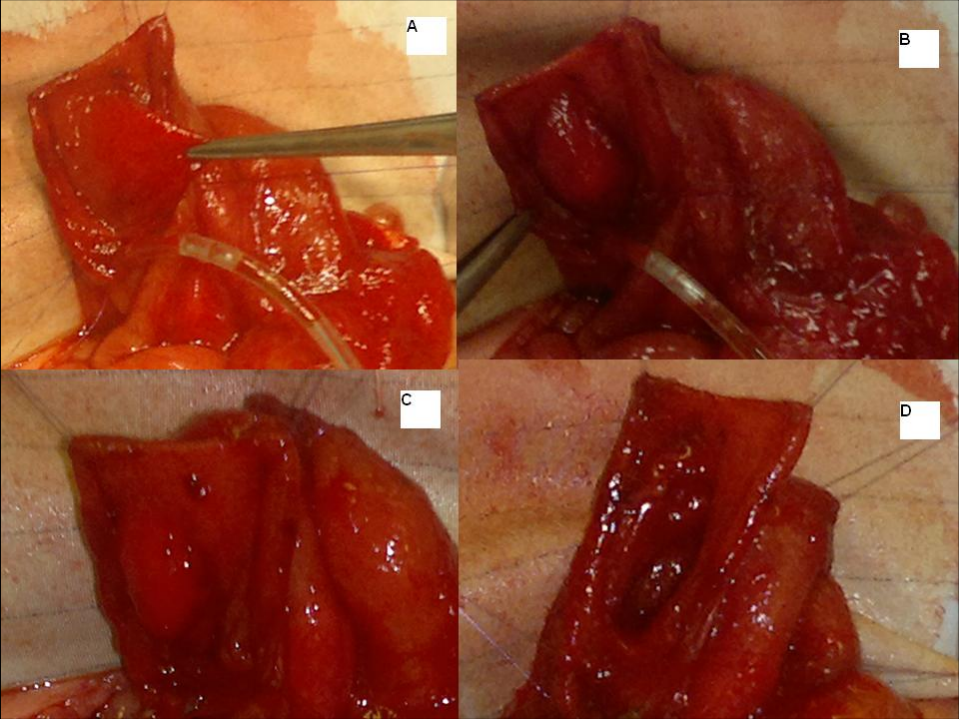
Figure 2: Intra-operative findings and steps.

Initially a small central hole was made; to see both sides of the diaphragm and the site of attachment to the duodenal wall especially to safeguard it from ampullary area in the diaphragm; followed by complete resection leaving a small thin rim close to the duodenal wall more on the medial side than the lateral aspect (Fig. 2C and 2D). The duodenal incision was closed transversely after advancing a jejunal feeding tube distally (Fig. 1f). Her post operative period was uneventful. Nasojejunal feeds were started on second postoperative day which were tolerated well. The gastro-oesophageal reflux was severe and she failed to gain weight initially and needed anti-reflux medications. She was discharged home on anti-reflux medications at 2 weeks of age. At 8-month follow-up, she was free of any reflux symptoms. The anti-reflux medications were stopped. She is now 15 months of age, asymptomatic and thriving well.

Type I duodenal atresia is an early embryonic event and should have been detected during 12 or 20th week antenatal scans. Polyhydramnios is associated with underlying congenital anomalies in over 50% of cases. Late pregnancy scan for polyhydramnios should have actively looked for evidence of bowel obstruction to rule out an organic cause. Although there is time pressure on discharge of mother and baby from the delivery suite after normal delivery, such cases should be observed till they tolerate feeds and pass meconium. Duodenal development and solid cord status is an early event in the embryogenesis and hence passage of meconium at birth is exceptional and invariably a very good clinical indicator of the underlying obstruction [2]. Congenital duodenal obstructions present with bilious vomiting and are relatively uncommon to be proximal to the ampulla of Vater, the most common site being just at the ampulla. In case of preampullary diaphragm, the presentation must be with non-bilious emesis as found in our case [3]. 

Plain radiographs demonstrate the double-bubble appearance with no distal gas. Upper gastrointestinal contrast studies may delineate the windsock deformity, but may not be able to show the site of attachment of the diaphragm. Sometimes there is an indentation externally to mark the site of attachment of the diaphragm or palpable scar tissue of the rim or annular pancreas around the site of stenosis. Passing a Fogarty or Foley catheter beyond the diaphragm and inflating balloon and withdrawing back may be helpful in the windsock deformity with a hole in the membrane but that option is not available in case of complete diaphragm. Endoscopic correction of incomplete duodenal diaphragm with or without windsock deformity is possible but in a complete diaphragm, it may be counterproductive and lead to serious complications [4]. Laparoscopy may have the same difficulties as it is difficult to palpate the rim and the soft indentation may require guiding the firm tube to enter in to the duodenum and proceed forward rather than curl back [5]. Duodenoplasty and diaphragm excision allowed minimal invasion and the trans-duodenoplasty feeding tube permitted early tube feeds to be started reducing the need for total parenteral nutrition. In conclusion, we believe that complete pre-ampullary duodenal membrane with windsock deformity could be a diagnostic and therapeutic challenge. Even in presence of polyhydramnios and modern prenatal screening programmes in the best centers, the prenatal diagnosis can be missed.

## Footnotes

**Source of Support:** Nil

**Conflict of Interest:** None
